# Data from proteome analysis of *Lasiodiplodia theobromae* (Botryosphaeriaceae)

**DOI:** 10.1016/j.dib.2017.04.058

**Published:** 2017-05-17

**Authors:** Carla C. Uranga, Majid Ghassemian, Rufina Hernández-Martínez

**Affiliations:** a Centro de Investigación Científica y de Educación Superior de Ensenada (CICESE), Carretera Ensenada-Tijuana 3918, Zona Playitas, 22860 Ensenada, B.C., Mexico; b University of California, San Diego, Department of Chemistry and Biochemistry, 9500 Gilman Drive, La Jolla, CA 92093-0378, United States

**Keywords:** Trunk-disease fungi, LC-nanoESI-MS, Proteomics, *de novo* peptide sequencing, Gene ontology

## Abstract

Trunk disease fungi are a global problem affecting many economically important fruiting trees. The Botryosphaeriaceae are a family of trunk disease fungi that require detailed biochemical characterization in order to gain insight into their pathogenicity. The application of a modified Folch extraction to protein extraction from the Botryosphaeriaceae *Lasiodiplodia theobromae* generated an unprecedented data set of protein identifications from fragmentation analysis and *de novo* peptide sequencing of its proteome. This article contains data from protein identifications obtained from a database-dependent fragmentation analysis using three different proteomics algorithms (MSGF, Comet and X! Tandem *via* the SearchGUI proteomics pipeline program) and *de novo* peptide sequencing. Included are data sets of gene ontology annotations using an all-Uniprot ontology database, as well as a *Saccharomyces cerevisiae*-only and a *Candida albicans*-only ontology database, in order to discern between those proteins involved in common functions with *S. cerevisiae* and those in common with the pathogenic yeast *C. albicans*. Our results reveal the proteome of *L. theobromae* contains more ontological categories in common to *C. albicans*, yet possesses a much wider metabolic repertoire than any of the yeasts studied in this work. Many novel proteins of interest were identified for further biochemical characterization and annotation efforts, as further discussed in the article referencing this article (1). Interactive Cytoscape networks of molecular functions of identified peptides using an all-Uniprot ontological database are included. Data, including raw data, are available *via* ProteomeXchange with identifier PXD005283.

**Specifications Table**

**Table:** 

Subject area	*Database-dependent peptide fragmentation protein identification and de novo sequencing of peptides from filamentous fungi*
More specific subject area	*Database-dependent peptide fragmentation-based protein identifications and de novo peptide sequencing of peptides from Lasiodiplodia theobromae*
Type of data	*SearchGUI protein identification data and DeNovoGUI peptide sequencing data*
How data was acquired	*Mass spectrometry with an AB SCIEX Triple TOF mass spectrometer*
Data format	*Protein identifications were analyzed and filtered with a <1% False Discovery Rate (FDR). De novo sequencing data reports the most homologous peptides in the Uniprot database to those from L. theobromae.*
Experimental factors	*Folch Extraction of triplicate incubations of L. theobromae in Vogel׳s salts supplemented with both 5% glucose and 5% grapeseed oil.*
Experimental features	*From previous work, it was shown that under these conditions, fatty acid esters with physiological activity in plants were detected and produced abundantly**[2]*.
Data source location	*Ensenada, Baja California, Mexico and San Diego, California, USA.*
Data accessibility	*The mass spectrometry proteomics data have been deposited to the ProteomeXchange Consortium via the PRIDE [3] partner repository with the dataset identifier*PXD005283.

**Value of the data**

• This is the first report of peptide fragmentation and *de novo* sequencing analysis of *Lasiodiplodia theobromae*, a pathogen of primarily economically important fruiting trees.
• Peptide fragmentation and *de novo* peptide sequencing analysis yielded many novel protein identifications that may aid in understanding fungal metabolism and further advance biochemical annotation efforts.
• The application of a Folch extraction is novel for filamentous fungi, and resulted in an extensive data set that may makes future proteomics experiments more efficient.
• Bioinformatics of filamentous fungi requires development, and this data is a step in that direction. Cytoscape networks are included for molecular function annotations for database-dependent protein identifications *via* fragmentation analysis and *de novo* peptide sequencing.

## 1 Data

The data consists in database-dependent peptide fragmentation analysis using the SearchGUI program [2] with the MSGF, Comet, and X!Tandem sequencing algorithms, identified with a 1% FDR. The entire list of protein identifications is available in this article as Supplementary data. Interactive Cytoscape networks are included, as well as full ontology reports using an all-Uniprot annotation database, as well as *Saccharomyces cerevisiae*-only and *Candida albicans*-only annotation databases. *De novo* peptide sequencing results are included in Supplementary data, as well as complete BLASTp protein identification results.

## 2 Experimental design, materials and methods

*L. theobromae* UCD256Ma (isolated in Madera County, California, USA) was provided by Dr. Douglas Gubler from the University of California at Davis [3], and incubated and extracted as described in the accompanying article [1]. Briefly, 0.5 g of the solids from the 50 mL fungal incubations (*L. theobromae* incubated in 5% glucose and 5% grapeseed oil and Vogel׳s salts for 20 days and lyophilized) remaining from the Folch extraction were dried under a stream of nitrogen and re-suspended in 50 mM Tris buffer, pH 8.00. Acetonitrile was added to the sample to a final concentration of 10%. The samples were then boiled for 5 min and cooled to room temperature. TCEP (Tris (2-carboxyethyl) phosphine) was added to 1 mM (final concentration) and the samples were incubated at 37 °C for 30 min. Subsequently, the samples were carboxymethylated with 0.5 mg/ml of iodoacetamide for 30 min at 37 °C in dark followed by neutralization with 2 mM TCEP (final concentration). Samples were boiled for 10 min followed by protease digestion with a 1:100 ratio of trypsin: protein (Pierce™ Trypsin Protease, MS Grade Catalog number: 90057 with K, R specificity). After an overnight digestion, samples were centrifuged on a desktop microfuge at max speed (15,000 rpm) for 10 min to remove the insoluble fraction. The soluble fraction was adjusted to 0.2% formic acid and 5% acetonitrile and its peptide content isolated using C-18 solid phase extraction (Thermo Scientific, PI-87782) as described by the manufacturer.

Proteomics mass spectrometry was done at the University of California, San Diego as described in the accompanying article [1]. The nano-spray ionization experiments were performed using a TripleTOF 5600 hybrid mass spectrometer (ABSCIEX) interfaced with a nano-scale reversed-phase UPLC (Waters nano ACQUITY) using a 20 cm to 75 μM ID glass capillary packed with 2.5-µM C18 (130) CSH^™^ beads (Waters). Peptides were eluted from the C18 column into the mass spectrometer with a linear gradient (5–80%) of acetonitrile (ACN) at a flow rate of 250 μL/min for 90 min. The buffers used to create the ACN gradient were Buffer A (98% H_2_O, 2% ACN, 0.1% formic acid and 0.005% TFA) and Buffer B (100% ACN, 0.1% formic acid, and 0.005% TFA). MS/MS data were obtained in a data-dependent manner in which the MS1 data was acquired for 250 ms at m/z of 400–1250 Da and the MS/MS data was acquired from m/z of 50 to 2000 Da. An MS1-TOF acquisition time of 250 ms was set, followed by 50 MS2 events of 48 ms acquisition time for each event. The threshold to trigger the MS2 event was set to 150 counts, when the ion had the charge state +2, +3 and +4. The ion exclusion time was set to 4 s.

## 3 Protein identification

This information appears as in the accompanying article [1]. This methodology is replicated in this article for the reader׳s convenience. Peak lists obtained from MS/MS spectra were identified using X! Tandem Vengeance (2015.12.15.2) [4], MS-GF+ version Beta (v10282) [5] and either OMSSA version 2.1.9 [6] or Comet version 2016.01 rev. 2 [7]. The search was conducted using SearchGUI version 3.1.2 [2]. The data was searched against a whole Uniprot/Swissprot database search (manually annotated and reviewed), [8] as well as a non-redundant Botryosphaeriaceae-only database downloaded from NCBI [9]. An all-human database from Uniprot was also used for further assessing protein identifications. All identification data from each database may be found as Supplementary Data S2, S3 and S5.

The identification settings were as follows: Trypsin with a maximum of 2 missed cleavages; 60.0 ppm as MS1 and 0.8 Da as MS2 tolerances; fixed modifications: Carbamidomethylation of C (+57.021464 Da) and Oxidation of M (+15.994915 Da), variable modifications: Acetylation of protein N-term (+42.010565 Da), Pyrolidone from E (+18.010565 Da), Pyrolidone from Q (+17.026549 Da) and Pyrolidone from carbamidomethylated C (+17.026549 Da). All algorithm- specific settings are listed in the Certificate of Analysis available in Supplementary Data S1.

Peptides and proteins were inferred from the spectrum identification results using PeptideShaker version 1.13.6 [10]. Peptide Spectrum Matches (PSMs), peptides and proteins were validated at a 1.0% False Discovery Rate (FDR) estimated using a decoy hit distribution. All validation thresholds are listed in the Certificate of Analysis and are available in Supplementary Data S1A, S1B, and S1C and for all databases searched. Post-translational modification localizations were scored using the D-score [11] and the A-score [12] with a threshold of 95.0 as implemented in the compomics-utilities package [13]. An A-score above 95.0 was considered a confident localization. The mass spectrometry data along with the identification results have been deposited to the ProteomeXchange Consortium [14] *via* the PRIDE partner repository [15] with the dataset identifier PXD005283.

Gene ontology (GO) analysis of enriched proteins was done on all those hits obtained from the Uniprot database [8]. The software Cytoscape [16] with the BiNGO plugin [17] was used for GO and enrichment analysis using up-to-date databases, applying a hypergeometric test with a significance level (*p*-value)<0.05, as well as a Benjamini and Hochberg false discovery rate (FDR) correction. Interactive Cytoscape BiNGO networks were created with data from all algorithms, and annotated with an all-Uniprot ontology database, available in Fig. 1 in this article. All gene ontology annotations may be found in Supplementary Data S5 and S6 in this article.

*De novo* peptide sequencing was performed in order to compare results and explore peptides *via* sequence homology with sequenced proteins found in the entire Uniprot database using BLAST. The program DeNovoGUI version 1.14.5 was used for this purpose [18], and both Novor [19] and PepNovo [20] were used for peptide sequencing. The mass allowance parameters were, for precursor mass tolerance: 10 ppm, and a fragment mass tolerance of 0.5 Da. Post-translational modification settings consisted in carbamidomethylation of cysteine (fixed) and oxidation of methionine (variable). All peptides were searched against the entire Uniprot database using a standalone version of NCBI-BLAST [21], with one peptide match per spectrum (most significant) and one BLAST match per peptide (most significant, lowest E-value). The BLAST match data was also analyzed for gene ontology (molecular functions) as described.

## Appendix A Supplementary material

Figure 1. Cytoscape BiNGO interactive networks of enriched gene ontology categories using protein identifications of an all-Swissprot (Uniprot) database search with combined protein identification data from MSGF, and X! Tandem, and OMSSA or Comet Algorithms (*via* SearchGUI), all identified with a 1% FDR and *de novo* peptide sequencing. A: Enriched molecular function ontology of proteins identified *via* fragmentation analysis using an all-Uniprot ontology database.

.

Figure 1. Cytoscape BiNGO interactive networks of enriched gene ontology categories using protein identifications of an all-Swissprot (Uniprot) database search with combined protein identification data from MSGF, and X! Tandem, and OMSSA or Comet Algorithms (*via* SearchGUI), all identified with a 1% FDR and *de novo* peptide sequencing. B: Enriched molecular function ontology of *de novo* peptide sequences using and all-Uniprot ontology database. Node size represents the number of genes assigned to each annotation, node color represents enrichment significance, with darker color shades indicating lower p-values (higher significance).

.

**Supplementary Data 2**: This data consists in SearchGUI output of a database-dependent fragmentation analysis. The first Excel sheet contains identified proteins whereas the second sheet contains a peptide sequence report from a search using the Botryosphaeriaceae-only NCBI database.

.

**Supplementary Data 4**: This data contains SearchGUI output of database-dependent fragmentation analysis. The first Excel sheet contains identified proteins whereas the second sheet contains a peptide sequence report from a search using the the Human-only Uniprot database.

.

**Supplementary Data 5**: This data includes a complete Cytoscape BiNGO gene ontology analysis, including biological processes (BP), molecular functions (MF), and cellular components (CC) for the proteome of *Lasiodiplodia theobromae* incubated in 5% grapeseed oil and 5% glucose compared to an all-Uniprot ontology database, a *Candida albicans*-only ontology database and a *Saccharomyces cerevisiae*-only ontology database.

.

**Supplementary Data 6**: This data comprises peptide sequences from *de novo* sequencing of the phytopathogen *Lasiodiplodia theobromae* using the NOVOR algorithm. The Excel file contains the following sheets: NOVOR algorithm output, BLASTp input, BLASTp search results, a Uniprot ID gene ontology (GO) search and Cytoscape BiNGO molecular function enrichment analysis. Datasets are arranged in order in separate Excel sheets.

## References

[1] Uranga C.C., Ghassemian M., Hernandez-Martinez R. (2017). Novel proteins from proteomic analysis of the trunk disease fungus Lasiodiplodia theobromae (Botryosphaeriaceae). Biochim. Open.

[2] Vaudel M., Barsnes H., Berven F.S., Sickmann A., Martens L. (2011). SearchGUI: an open-source graphical user interface for simultaneous OMSSA and X!Tandem searches. Proteomics.

[3] Úrbez-Torres J.R., Leavitt G.M., Voegel T.M., Gubler W.D. (2006). Identification and distribution of Botryosphaeria spp. Associated with grapevine cankers in California. Plant Dis..

[4] Craig R., Beavis R.C. (2004). TANDEM: matching proteins with tandem mass spectra. Bioinformatics.

[5] Kim S., Pevzner P.A. (2014). MS-GF+ makes progress towards a universal database search tool for proteomics. Nat. Commun. [Internet] Nat. Publ. Group.

[6] Geer L.Y., Markey S.P., Kowalak J.A., Wagner L., Xu M., Maynard D.M. (2004). Open mass spectrometry search algorithm research articles. J. Proteome Res..

[7] Eng J.K., Jahan T.A., Hoopmann M.R. (2013). Comet: an open-source MS/MS sequence database search tool. Proteomics.

[8] Bateman A., Martin M.J., O’Donovan C., Magrane M., Apweiler R., Alpi E. (2015). UniProt: a hub for protein information. Nucleic Acids Res..

[9] Geer L.Y., Marchler-Bauer A., Geer R.C., Han L., He J., He S. (2009). The NCBI BioSystems database. Nucleic Acids Res.

[10] Vaudel M., Burkhart J.M., Zahedi R.P., Oveland E., Berven F.S., Sickmann A. (2015). PeptideShaker enables reanalysis of MS-derived proteomics data sets. Nat. Biotechnol..

[11] Vaudel M., Breiter D., Beck F., Rahnenführer J., Martens L., Zahedi R.P. (2013). D-score: a search engine independent MD-score. Proteomics.

[12] Beausoleil S.A., Villén J., Gerber S.A., Rush J., Gygi S.P. (2006). A probability-based approach for high-throughput protein phosphorylation analysis and site localization. Nat. Biotechnol..

[13] Barsnes H., Vaudel M., Colaert N., Helsens K., Sickmann A., Berven F.S. (2011). compomics-utilities: an open-source Java library for computational proteomics. BMC Bioinform. [Internet] Biomed. Cent. Ltd..

[14] Vizcaíno J., Deutsch E.E.W., Wang R., Vizcaino J.A., Deutsch E.E.W., Wang R. (2014). ProteomeXchange provides globally coordinated proteomics data submission and dissemination. Nat. Biotechnol..

[15] Martens L., Hermjakob H., Jones P., Adamsk M., Taylor C., States D. (2005). PRIDE: the proteomics identifications database. Proteomics.

[16] Christmas R., Avila-Campillo I., Bolouri H., Schwikowski B., Anderson M., Kelley R. (2005). Cytoscape: a software environment for integrated models of biomolecular interaction networks. Am. Assoc. Cancer Res Educ. B.

[17] Maere S., Heymans K., Kuiper M. (2005). BiNGO: a Cytoscape plugin to assess overrepresentation of Gene Ontology categories in Biological Networks. Bioinformatics.

[18] Muth T., Weilnböck L., Rapp E., Huber C.G., Martens L., Vaudel M. (2014). DeNovoGUI: an open source graphical user interface for de novo sequencing of tandem mass spectra. J. Proteome Res..

[19] Ma B. (2015). Novor: real-time peptide de Novo sequencing software. J Am. Soc. Mass Spectrom..

[20] Frank A., Pevzner P. (2005). PepNovo: de novo peptide sequencing via probabilistic network modeling. Anal. Chem..

[21] Camacho C., Coulouris G., Avagyan V., Ma N., Papadopoulos J., Bealer K. (2009). BLAST+: architecture and applications. BMC Bioinform..

